# Vertebral hemangiomas: a review on diagnosis and management

**DOI:** 10.1186/s13018-024-04799-5

**Published:** 2024-05-24

**Authors:** Kyle Kato, Nahom Teferi, Meron Challa, Kathryn Eschbacher, Satoshi Yamaguchi

**Affiliations:** 1 University of Iowa Carver, College of Medicine, Iowa City, IA USA; 2 Department of Neurosurgery, University of Iowa Carver, College of Medicine, Iowa City, IA USA; 3 Department of Pathology, University of Iowa Carver, College of Medicine,, Iowa City, IA USA

**Keywords:** Vertebral hemangioma, Laminectomy, Fusion, Sclerotherapy, Vertebroplasty, Radiotherapy

## Abstract

**Background:**

Vertebral hemangiomas (VHs) are the most common benign tumors of the spinal column and are often encountered incidentally during routine spinal imaging.

**Methods:**

A retrospective review of the inpatient and outpatient hospital records at our institution was performed for the diagnosis of VHs from January 2005 to September 2023. Search filters included “vertebral hemangioma,” "back pain,” “weakness,” “radiculopathy,” and “focal neurological deficits.” Radiographic evaluation of these patients included plain X-rays, CT, and MRI. Following confirmation of a diagnosis of VH, these images were used to generate the figures used in this manuscript. Moreover, an extensive literature search was conducted using PubMed for the literature review portion of the manuscript.

**Result:**

VHs are benign vascular proliferations that cause remodeling of bony trabeculae in the vertebral body of the spinal column. Horizontal trabeculae deteriorate leading to thickening of vertical trabeculae which causes a striated appearance on sagittal magnetic resonance imaging (MRI) and computed tomography (CT), “Corduroy sign,” and a punctuated appearance on axial imaging, “Polka dot sign.” These findings are seen in “typical vertebral hemangiomas” due to a low vascular-to-fat ratio of the lesion. Contrarily, atypical vertebral hemangiomas may or may not demonstrate the “Corduroy” or “Polka-dot” signs due to lower amounts of fat and a higher vascular component. Atypical vertebral hemangiomas often mimic other neoplastic pathologies, making diagnosis challenging. Although most VHs are asymptomatic, aggressive vertebral hemangiomas can present with neurologic sequelae such as myelopathy and radiculopathy due to nerve root and/or spinal cord compression. Asymptomatic vertebral hemangiomas do not require therapy, and there are many treatment options for vertebral hemangiomas causing pain, radiculopathy, and/or myelopathy. Surgery (corpectomy, laminectomy), percutaneous techniques (vertebroplasty, sclerotherapy, embolization), and radiotherapy can be used in combination or isolation as appropriate. Specific treatment options depend on the lesion's size/location and the extent of neural element compression. There is no consensus on the optimal treatment plan for symptomatic vertebral hemangioma patients, although management algorithms have been proposed.

**Conclusion:**

While typical vertebral hemangioma diagnosis is relatively straightforward, the differential diagnosis is broad for atypical and aggressive lesions. There is an ongoing debate as to the best approach for managing symptomatic cases, however, surgical resection is often considered first line treatment for patients with neurologic deficit.

## Introduction

Vertebral hemangiomas (VHs) are benign vascular lesions formed from vascular proliferation in bone marrow spaces that are limited by bony trabeculae [1]. VHs are quite common and are often incidental findings on spinal computed tomography (CT) and magnetic resonance imaging (MRI) of patients presenting with back or neck pain [2, 3]. Previous, large autopsy series such as Schmorl (1926) and Junghanns (1932) found a VH prevalence of 11% in adult specimens [1, 4]. However, the prevalence is believed to be higher as modern imaging techniques allow for better detection of small VHs that may not be easily diagnosed on autopsy specimens [5]. They can occur at any age but are most often seen in individuals in their 5th decade of life with a slight female preponderance [2, 6, 7]. Most VHs are found in the thoracic or lumbar spinal column and often involve the vertebral body, though they can extend to the pedicle, lamina, or spinous process, and may span multiple spinal segments [5].

The vast majority of VHs are asymptomatic, quiescent lesions [3]. Prior studies have stated less than 5% of VHs are symptomatic [8, 9], although the 2023 study by Teferi et. al. demonstrated 35% of their 75 VH patients presented with symptoms including localized pain, numbness, and/or paresthesia [1]. 85% of symptomatic cases in this series were found to have VHs localized in the thoracic spine [1].

Among symptomatic VHs, up to 20–45% of cases may exhibit aggressive features including damage to surrounding bone and soft tissue or demonstrate rapid growth that extends beyond the vertebral body and invades the paravertebral and/or epidural space [1, 5, 10, 11]. When “aggressive”, VHs may compress the spinal cord and nerve roots causing severe symptoms [1, 5]. 45% of symptomatic VH patients present with neurologic deficits secondary to compressive lesions, bony expansion, disrupted blood flow, or vertebral body collapse while the remaining 55% present solely with back pain [8, 12–15].

VHs are primarily diagnosed with radiographs, CT, and MRI, although other studies such as angiography, nuclear medicine studies, and positron emission—computed tomography (PET-CT) have been previously utilized to a lesser extent [1, 15–19]. Radiologically, these lesions can be grouped into Typical, Atypical, and Aggressive subtypes (see radiological features). Histologically, VHs are composed of varying proportions of adipocytes, blood vessels, and interstitial edema which leads to thickening of vertical trabeculae in the affected vertebra [5]. This histopathology leads to the characteristic “polka-dot” sign on axial CT/MRI and “corduroy” sign on coronal and sagittal CT/MRI [5, 20].

In terms of management, conservative treatment with observation and pain control are the mainstay of treatment for asymptomatic VH patients and those with mild-to-moderate pain respectively [21]. Surgical decompression is indicated for patients with neurologic deficits including compressive myelopathy or radiculopathy [22]. Other symptomatic patients have a wide variety of treatment options available including sclerotherapy, embolization, radiotherapy, and/or vertebroplasty [1, 5, 23]. The best approach in managing an individual patient with a symptomatic VH has not been elucidated and there have been different management algorithms suggested based on varying institutional experiences [1, 5, 24, 25].

This article will review what is currently known regarding VHs. Diagnostic techniques and challenges will be highlighted as well as current treatment recommendations from the literature.

## Methods

A retrospective review of the inpatient and outpatient hospital records at our institution was performed for the diagnosis of VHs from January 2005 to September 2023. Search filters included “vertebral hemangioma” "back pain,” “weakness,” “radiculopathy,” and “focal neurological deficits.” Radiographic evaluation of these patients included plain X-rays, CT, and MRI. Following confirmation of a diagnosis of VH, these images were used to generate the figures used in this manuscript. Moreover, an extensive literature search was conducted using PubMed for the literature review portion of the manuscript.

## Results

68 Articles were selected from our PubMed search. This article will review what is currently known about VHs. Diagnostic techniques and challenges will be highlighted as well as current treatment recommendations from the literature.

### Histopathological features

VHs are benign tumors composed of various sized blood vessels, adipocytes, smooth muscle, fibrous tissue, hemosiderin, interstitial edema, and remodeled bone [5, 7, 26, 27]. Macroscopically, they appear as soft, well-demarcated, dark red masses with intralesional, sclerotic boney trabeculae and scattered blood-filled cavities lending to a honeycomb appearance [5–7].

**Fig. 1:**
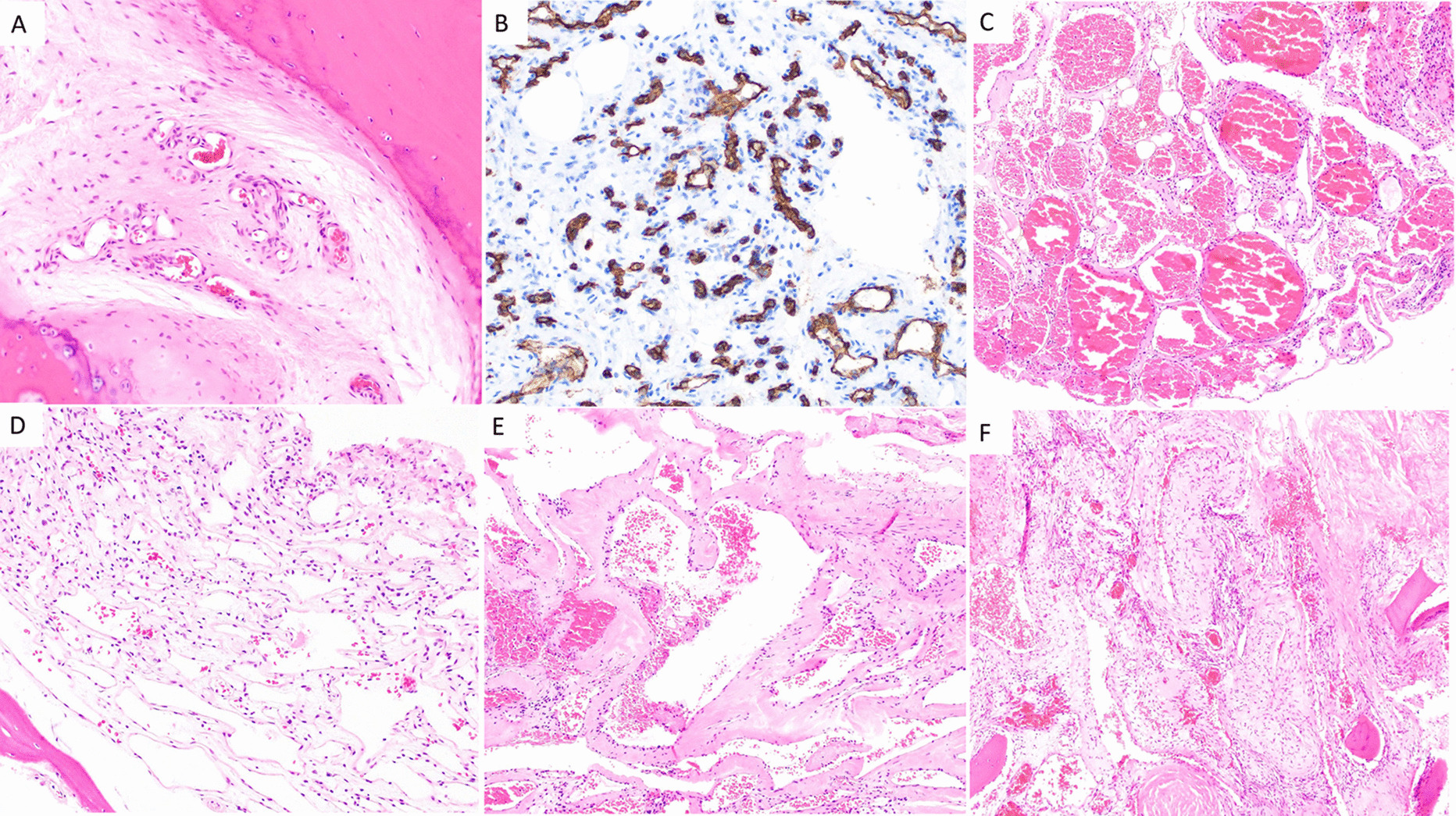
Capillary hemangioma (**A** and **B**): **A** H&E 200× magnification showing proliferation of small caliber vessels within a fibrous stroma with surrounding bone, **B** CD34 immunohistochemical stain, 200× magnification highlighting small caliber vascular spaces. Cavernous hemangioma (**C** and **D**): **C** H&E 100× magnification showing proliferation of thin-walled, dilated, blood filled vascular channels, **D** H&E 200× magnification: Thin-walled, dilated vascular channels within a loose stroma with adjacent mature bone. Venous hemangioma (**E** and **F**): **E** H&E 100**× **magnification showing abnormal proliferation of thick-walled vessels with dilated lumens. **F** H&E 100× magnification reveals tightly packed, thick-walled vessels with adjacent fragments of mature bone

Microscopically, there are four subtypes of hemangiomas based on vascular composition: capillary, cavernous, arteriovenous (AV), and venous hemangiomas [28] (Fig. 1). Capillary hemangiomas are composed of small, capillary-sized blood vessels while cavernous hemangiomas present with collections of larger, dilated blood vessels [1]. AV hemangiomas are composed of interconnected arterial and venous networks while an abnormal collection of veins comprises venous hemangiomas [1]. VHs are predominately capillary and cavernous subtypes with thin-walled blood vessels surrounded by edematous stroma and boney trabeculae that permeate the bone marrow space [1, 7, 27]. In a sample of 64 surgically treated VHs cases, Pastushyn et al. reported 50% were capillary subtype, 28% were cavernous subtype, and 22% were mixed [29]. Occasionally, secondary reactive phenomena such as fibrous and/or adipose involution of bone marrow and remodeling of bone trabeculae may be seen [7, 26]. Symptomatic VHs can be caused by all hemangioma subtypes, and there are no distinguishing features between subtypes on imaging [1]. However, cavernous and capillary subtypes are associated with favorable postsurgical outcomes [29].

### Radiographic features

The histopathology of VHs gives rise to imaging features used to classify VHs as typical, atypical, or aggressive [13]. Typical and atypical MRI findings are correlated with the intralesional ratio of fat to vascular components [20]. Lesions with a high fat content are more likely to demonstrate features of typical VHs while those with a high vascular content (atypical VHs) tend to present without these findings [5, 30, 31]. Aggressive VHs have features including destruction of the cortex, invasion of the epidural and paravertebral spaces, and lesions extending beyond the vertebral body [13, 15, 20].

Laredo et al. demonstrated that VHs with a higher fatty content are generally quiescent lesions, while those with a higher vascular content are more likely to display “active” behavior and potentially evolve into compressive lesions [20]. Therefore, asymptomatic VHs can display both typical or atypical imaging findings while symptomatic lesions are more likely to present with atypical or aggressive findings [1]. Despite radiographically typical VHs being relatively easy to diagnose, atypical and aggressive VHs are much more challenging to recognize as they do not present with classic imaging findings and often mimic other pathologies such as multiple myeloma, metastatic bone lesions, and inflammatory conditions [5, 30, 31]. Compressive VHs often have coinciding radiologic and clinical classifications due to the correlation between aggressive behavior and compressive symptoms [5].

While MRI, CT, and radiographs are the primary imaging modalities used in the workup of VHs, other studies have also been used. Angiography will occasionally be performed to identify feeding/draining vessels and evaluate the blood supply to the spinal cord [5]. Multiphase technetium 99-methyl diphosphonate (^99^Tc-MDP) bone scintigraphy may show increased tracer uptake in all phases (perfusion, blood pool, and delayed) due to technetium 99-labeled red blood cell accumulation in the tumors, which occurs in all hemangiomas [16]. PET-CT has been used to classify VHs as “hot” or “cold” lesions based on the degree of 18-FDG and 68-Ga DOTATATE uptake [17–19]. Although angiography is useful in clarifying the vascular network of aggressive VHs primarily, nuclear medicine studies offer a much more limited contribution to diagnosis when compared to CT and MRI [5].

### Typical VHs

The collection of thin-walled, blood-filled spaces that comprise VHs cause resorption of horizontal trabeculae and reinforcement of vertical trabeculae, leading to a pattern of thickened vertical trabeculae interspersed with lower density bone of the nonexpanding vertebral body [15, 31, 32]. This composition is responsible for the “corduroy cloth” appearance seen in typical VHs on radiographic images [31].

**Fig. 2:**
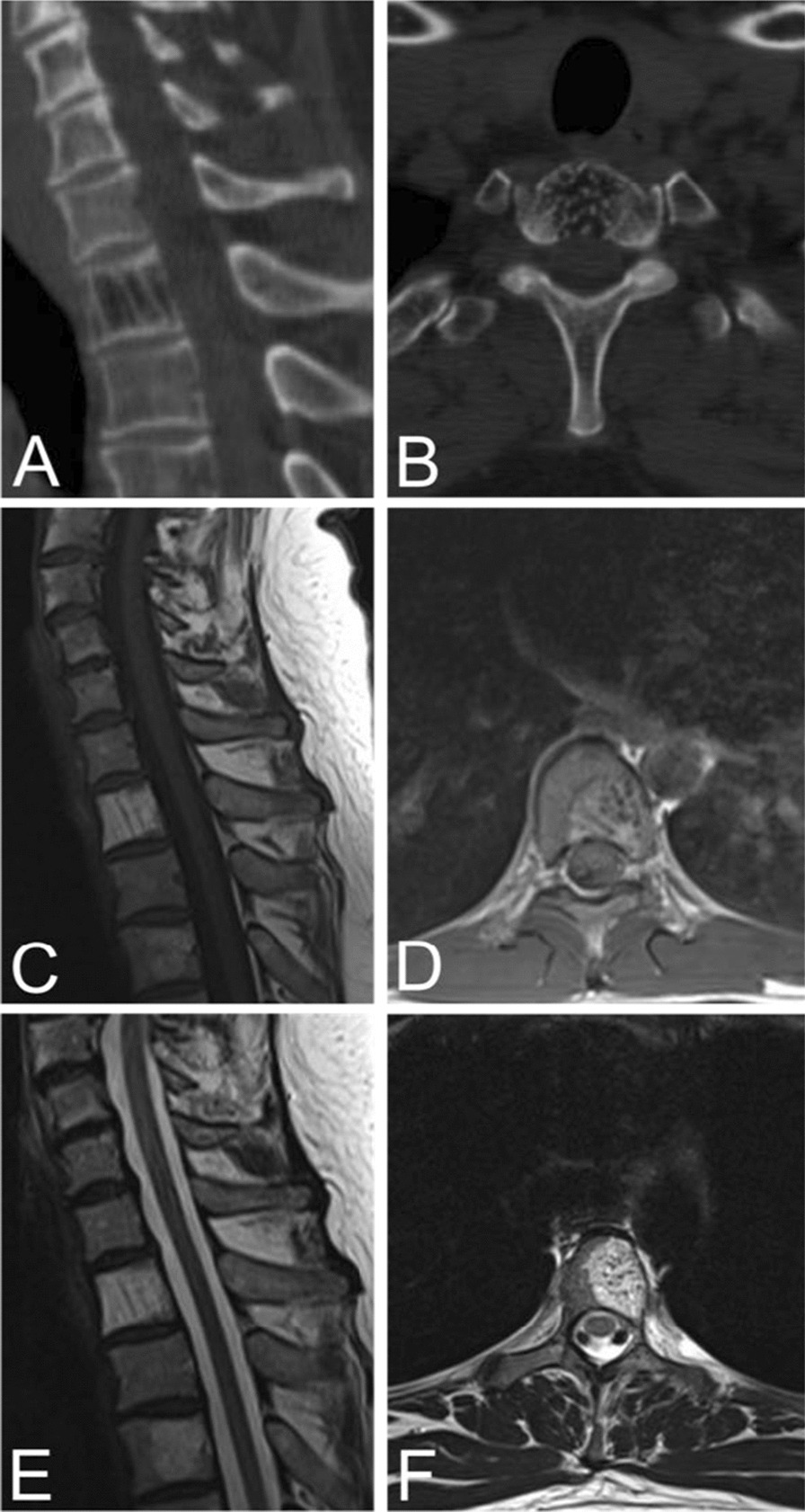
Sagittal (**A**) and axial (**B**) CT scans of a typical VH in an asymptomatic 50-year-old male demonstrating the “Corduroy” and “Polka-dot” signs respectively. Sagittal (**C**) and axial (**D**) T1-weighted MRIs of typical VHs are predominately hyperintense with areas of hypo-intensity due to thickening of vertical trabeculae. Sagittal (**E**) and axial (**F**) T2-weighted MRIs of typical VHs also appear as hyperintense lesions with areas of hypo-intensity that may demonstrate the “Corduroy” and “Polka-dot” signs as seen in CT images of typical VHs

On unenhanced axial CT images, typical VHs are characterized by a “polka dot” appearance, termed polka-dot sign. This is caused by small, punctate areas of high attenuation from hyperdense trabeculae surrounded by hypodense stroma [20, 33] (Fig. 2). Like radiographs, sagittal and coronal CT images display the “corduroy” sign caused by thickened trabeculae in a field of hypodense bone (Fig. 2). There is no extraosseous extension of the hemangioma in typical VHs [5].

**Fig. 3:**
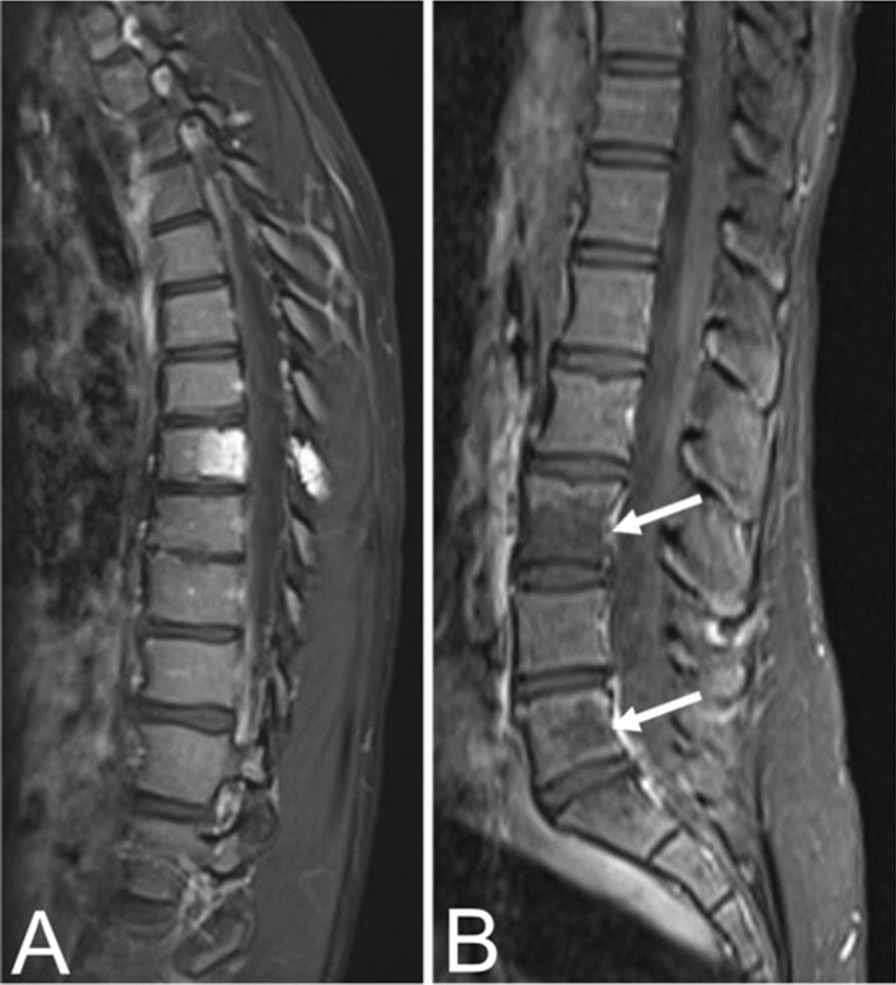
Contrast-enhanced T1 MRIs of a T8 VH in an asymptomatic fourteen-year-old female (**A**) and L3, L5 VHs in a thirty-one-year-old female with back pain (**B**), illustrating the heterogenous presentation of hemangiomas on post-contrast MRI

Typical VHs tend to appear as hyperintense lesions on T1- and T2-weighted MRI sequences due to predominately fatty overgrowth with penetrating blood vessels [31] (Fig. 2). There are punctate areas of slight hypointensity within the lesion on axial T1-weighted MRI due to thickened vertical trabeculae which resembles the “polka-dot" sign [5] (Fig. 2). These trabeculae appear as linear striations on sagittal/coronal T1- and T2-weighted MRI [5] (Fig. 2). Fluid-sensitive sequences (i.e. short-tau inversion recovery or fat-saturated T2-weighted MRI) appear slightly hyperintense due to the vascular components of the lesion, and T1-weighted MRI with contrast demonstrates heterogenous enhancement of the lesion [3] (Fig. 3).

### Atypical VHs

**Fig. 4:**
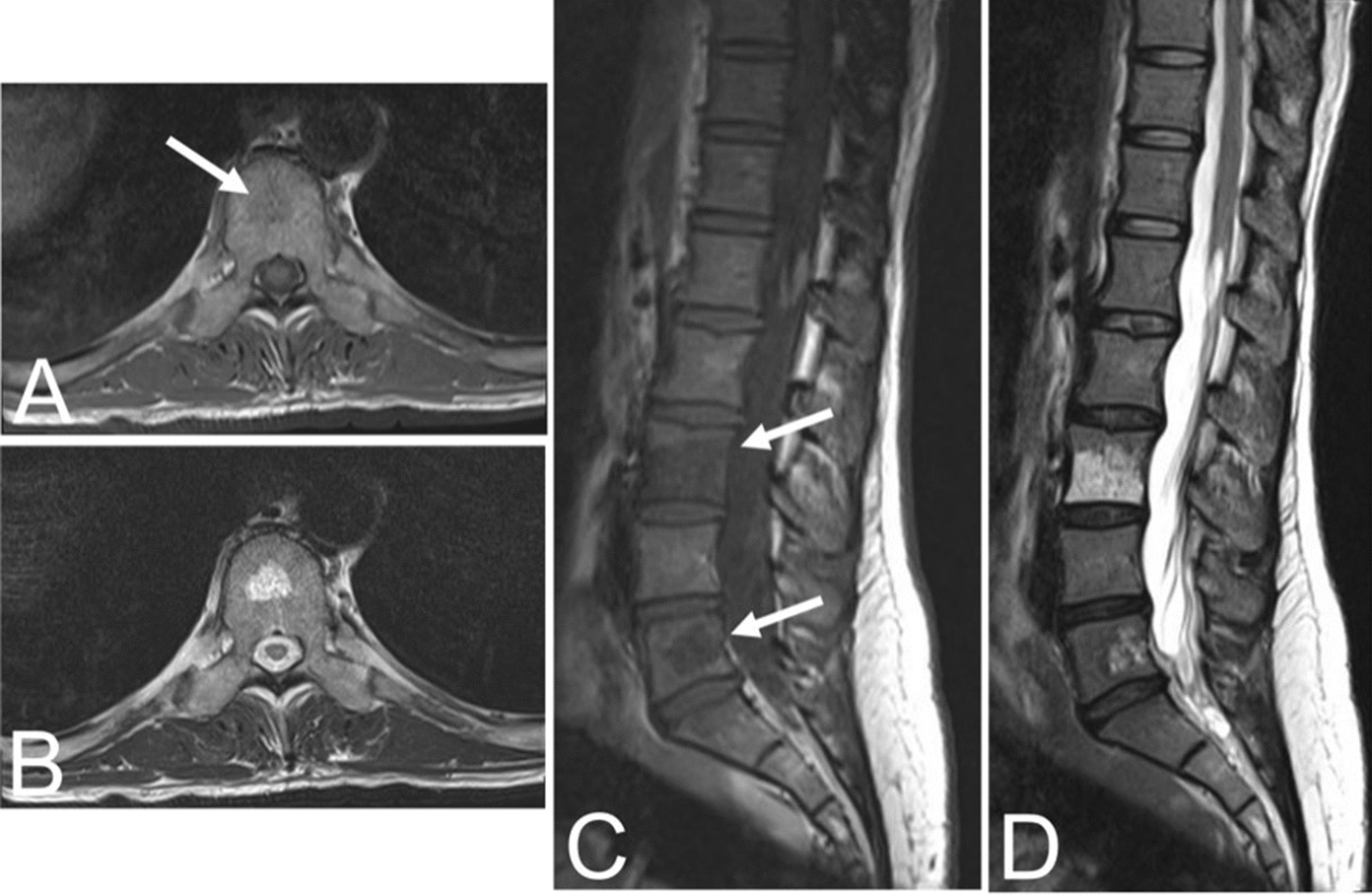
Asymptomatic fifty-six-year-old male with a T9 atypical vertebral hemangioma that appears iso- to hypointense on axial T1 MRI (**A**) and hyperintense on axial T2 MRI (**B**). Atypical vertebral hemangiomas of the L3 and L5 vertebral bodies in a thirty-one-year-old female who presented with backpain. Sagittal T1 (**C**) and T2 (**D**) demonstrate hypo- and hyperintense lesions respectively

In contrast to typical VHs, atypical VHs tend to have a higher vascular component-to-fat ratio and may not demonstrate the classical imaging findings such as the “corduroy” and “polka-dot” signs [5]. This composition gives the lesion an iso- to hypointense appearance on T1-weighted MRI as well as a very high intensity appearance on T2-weighted and fluid-sensitive MRI [20, 31] (Fig. 4). Atypical VHs often mimic primary bony malignancies or metastases and are more likely to demonstrate aggressive features, often making them difficult to diagnose [12–15].

### Aggressive VHs

**Fig. 5:**
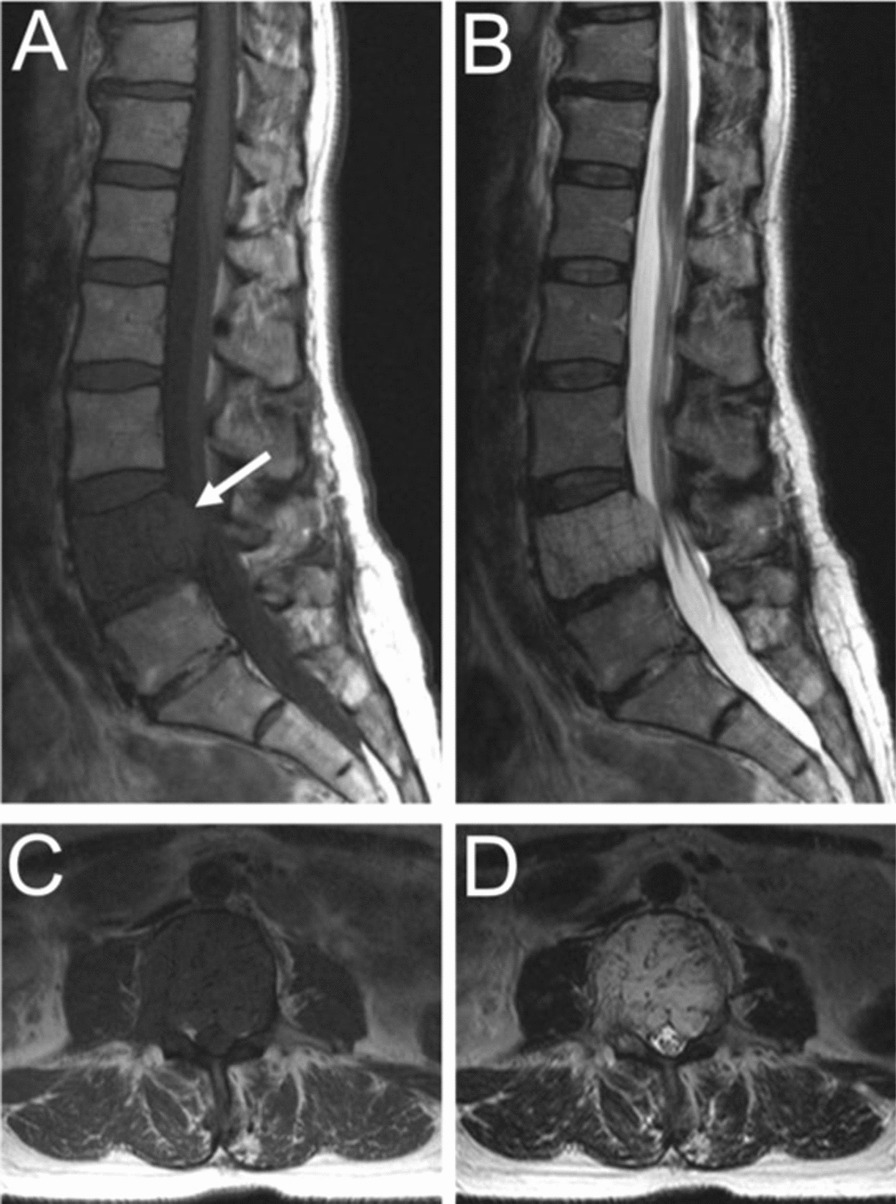
Fifty-five-year-old female with an aggressive vertebral hemangioma of the L4 vertebral body with extension into the spinal canal. **A** Sagittal T1 MRI shows hypo-intensity of the entire vertebral body, although vertebral height is maintained. **B** Sagittal T2 MRI redemonstrates the lesion but appears hyperintense due to the vascularity of the hemangioma. Axial T1 (**C**) and T2 (**D**) MRI show involvement of the pedicles bilaterally and extension of the lesion into the anterior epidural space

Aggressive VHs routinely have atypical features on any imaging modality [1, 5]. They may appear radiographically normal or show nonspecific findings such as osteoporosis, pedicle erosion, cortex expansion, vertebral collapse, or irregular vertical trabeculae associated with lytic areas of varying size [13, 15] (Fig. 5).

CT findings are often nonspecific, including features such as extraosseous soft tissue expansion, cortical ballooning, or cortical lysis [34, 35]. As with atypical VHs, the “corduroy” and “polka-dot” signs may not be readily visualized in aggressive or destructive lesions due to the higher vascular-to-fat ratio common in these hemangiomas [5]. However, it is important to be mindful of these signs because they can guide to the correct diagnosis. Other CT features that may assist in the diagnosis of inconspicuous VHs include extension of the lesion into the neural arch, involvement of the entire vertebral body, or an irregular honeycomb pattern due to serpentine vascular channels and fatty proliferation within the network of reorganizing bony trabeculae [20]. Vertebral fractures are rare due to the reinforcement of vertical trabeculae [1].

The composition of aggressive VHs, with a hypervascular stroma and less fat, results in a hypointense lesion on T1-weighted MRI [20, 31] (Fig. 5). Again, this may conceal the “corduroy” and “polka-dot” signs which remain amongst the most useful imaging findings in the diagnosis of VHs, particularly in cases where other findings are nonspecific [5]. These non-specific findings may include hyperintensity on T2-weighted MRI due to the vascular components of the lesion (Fig. 5), which is also seen in most neoplastic and inflammatory lesions [31]. Areas of hyperintensity on fluid-sensitive MRI and the presence of lipid-dense content within the lesion may be seen as well [31, 36]. Other features suggestive of an aggressive VH include a maintained vertebral body height, a sharp margin with normal marrow, an intact cortex adjacent to a paraspinal mass, or enlarged paraspinal vessels, however these findings are also nonspecific and relatively uncommon [5, 13]. Although highly unusual, there have been cases of aggressive VHs with extensive intraosseous fatty stroma and simultaneous extraosseous extension of the lesion, permitting a straightforward diagnosis [36].

Even though some aggressive VHs may be diagnosed on CT and MRI, challenging cases may warrant the use of more advanced imaging techniques for accurate diagnosis. Higher fluid content relative to cellular soft tissue gives hemangiomas a bright appearance on diffusion weighted imaging (DWI) with elevated apparent diffusion coefficient (ADC) values, distinguishing them from metastases [37]. Volume transfer constant (K^trans^) and plasma volume, which reflect capillary permeability and vessel density respectively, are quantitative measures derived from dynamic contrast enhanced magnetic resonance imaging (DCE MRI) perfusion imaging that can also be used to differentiate VHs and metastases [38]. K^trans^ and plasma volume are both low in VHs and elevated in metastatic lesions [38]. Furthermore, aggressive VHs may show a signal drop when comparing non-contrast T1-weighted MRI with and without fat suppression, as well as microscopic lipid content on chemical shift imaging [39]. Finally, characteristic findings of aggressive VHs in angiography include vertebral body arteriole dilation, multiple capillary phase blood pools, and complete vertebral body opacification [15].

Laredo et al. [15] proposed a six-point scoring system to assist in the diagnosis of aggressive VHs based on the more common features observed in radiographs and CT. One point was given for each of the following findings: a soft tissue mass, thoracic location between T3–T9, involvement of the entire vertebral body, an irregular honeycomb appearance, cortical expansion, and extension into the neural arch [15]. The authors suggest that aggressive VHs should be suspected when a patient presents with nerve root pain in association with three or more of these features [15]. However, additional studies are needed to determine the utility of this scoring system as the predictive power has not been determined [5].

Some VHs are difficult to diagnose because they can have nonspecific findings on radiographs, CT, and MRI, making characteristic findings such as the “corduroy” and “polka-dot” signs, when present, important diagnostic features. VHs may also coexist with other vertebral lesions, further complicating the diagnosis. In these cases, angiography can differentiate a VH from a nonvascular lesion [40]. Ultimately, a biopsy may be required for accurate diagnosis, especially when there is potential for a malignant lesion such as angiosarcoma or epithelioid hemangioendothelioma.

### Clinical features

VHs are often noted incidentally on spinal imaging and are often observed in patients in their fifth to sixth decade of life. Studies have shown that vertebral hemangiomas exhibit a slight female preponderance, with a male-to-female ratio of 1:1.5. [6]. Clinically, most VHs are asymptomatic and quiescent lesions, which rarely demonstrate active behavior and become symptomatic [41]. VHs occur most frequently in the thoracic spine [42], followed by the lumbar spine and cervical spine; sacral involvement is very rare [43].

When symptomatic, VHs can present with localized back pain or result in neurologic symptoms that are attributable to spinal cord compression, nerve root compression, or both, leading to myelopathy and/or radiculopathy [1]. At least 4 mechanisms of spinal cord and nerve root compression have been suggested: (1) hypertrophy or ballooning of the posterior cortex of the vertebral body caused by the angioma, (2) extension of the angioma through the cortex into the epidural space, (3) compression fracture of the involved vertebra, and (4) epidural hematoma [44]. When aggressive and symptomatic with spinal cord compression, VHs tend to occur in the thoracic spine [42].

Boriani et al. classified VHs into 4 groups based on the presence of symptoms and radiographic findings [45]. These include: Type I—latent, mild bony destruction with no symptoms; Type II—active, bony destruction with pain; Type III—aggressive, asymptomatic lesion with epidural and/or soft-tissue extension; and Type IV—aggressive, neurologic deficit with epidural and/or soft tissue extension.

### Management options

Most VHs are asymptomatic and do not require treatment [1, 21]. Treatment is indicated in cases with back pain or neurological symptoms, including myelopathy and/or radiculopathy, often caused by neuronal compression or vertebral fracture [1]. Previously, surgery was the primary treatment option offered to these patients, which was associated with an increased risk of complications, particularly intraoperative bleeding [1]. New modalities such as vertebroplasty have since gained traction as adjuncts or alternatives to surgery [1]. Today, there are several management options available for the treatment of symptomatic VHs, including conservative medical therapy, surgery, percutaneous techniques, radiotherapy, or a combination of these modalities [1, 46].

**Fig. 6:**
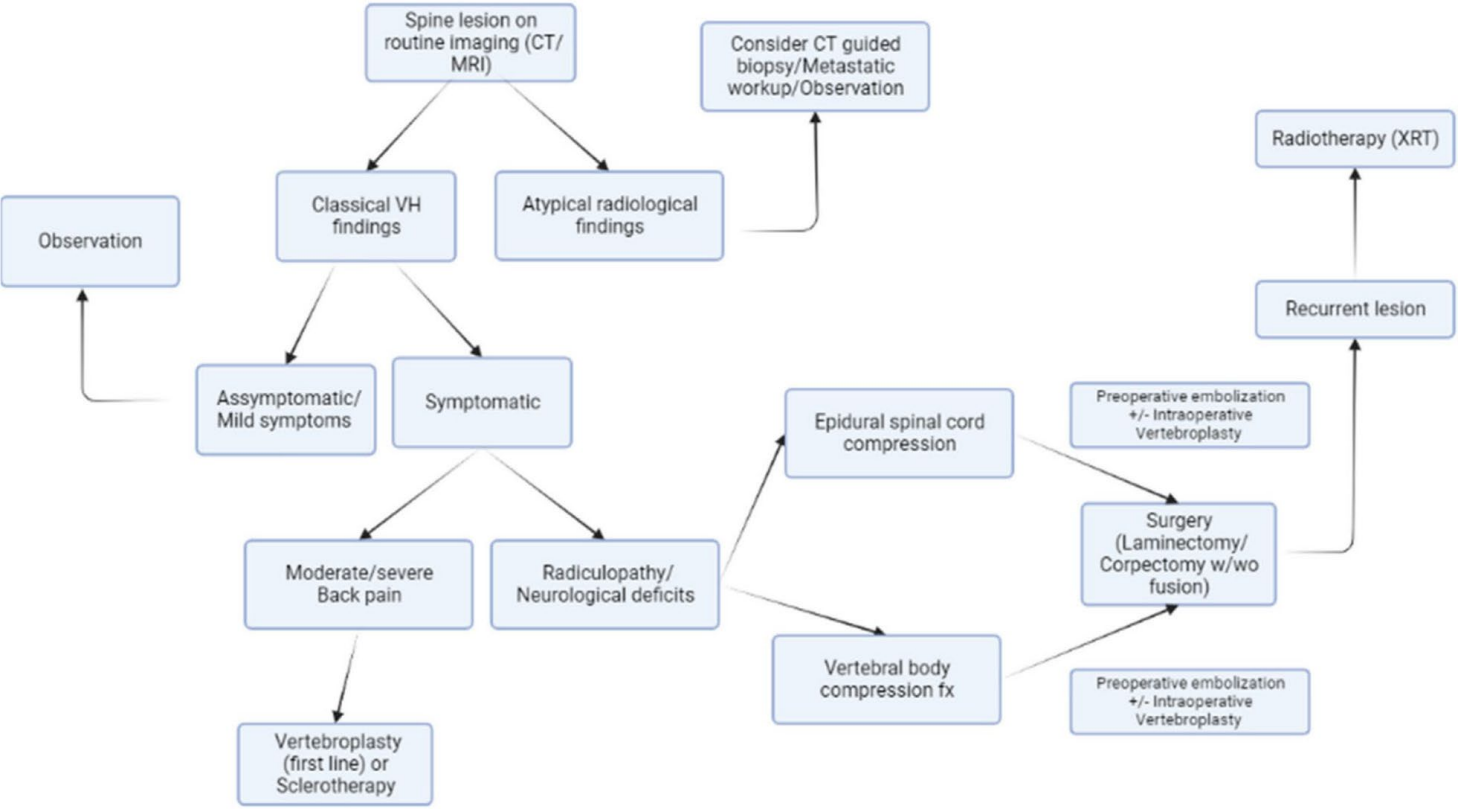
Algorithm for diagnosis and management of VHs proposed by Teferi et al. [1]

There is no consensus on the best treatment strategy, however recently Teferi et. al. proposed a treatment algorithm for VHs based on their institutional experience and literature review (Fig. 6) [1]. They recommend conservative management for typical, asymptomatic VHs, CT-guided biopsy and metastatic workup with PET-CT for radiographically atypical VHs, surgical intervention with or without adjuvant therapy in cases with epidural spinal cord compression or vertebral compression fracture, and radiotherapy for recurrent, asymptomatic VHs following surgery.

### Surgery

Surgical treatment of VHs is recommended in cases with rapid or progressive neurologic symptoms including compressive myelopathy or radiculopathy [47]. Baily et al. documented the first case of surgical management for VHs after they successfully resolved a patient’s paraplegia secondary to an aggressive VH [48]. Prior to the 1960s, the average neurological recovery rate was 73% (range, 43–85%) with a mortality rate of 11.7% [49]. This is consistent with a series published by Ghormley et al. in 1941 where 5 symptomatic VH patients were treated with decompressive laminectomy and postoperative radiotherapy. Although three patients achieved partial or complete resolution of neurologic deficits, the procedure resulted in the death of the remaining two patients secondary to significant blood loss [50]. There were very few cases of symptomatic VHs documented prior to the 1960s, with one literature review reporting only 64 instances of VHs with neurologic dysfunction [49]. More recent studies demonstrate improvement in surgical outcomes with neurological recovery reaching 100% and mortality as low as 0% [42].

The goal of surgery is to decompress neural elements and stabilize the spine [1]. Potential options include corpectomy, involving resection of a portion of the vertebral body containing the hemangioma, followed by anterior column reconstruction and/or laminectomy, which offers indirect decompression [1]. The selected approach depends on the size of the hemangioma and the extent of vertebral body and/or neural arch involvement due to potential weaknesses in the anterior column and the location of the epidural intrusion into the spinal canal [1]. For example, corpectomy and reconstruction could be performed in cases with ventral spinal cord compression while cases with dorsal compression could be treated with laminectomy [1].

Corpectomy has an increased risk of substantial intraoperative blood loss, up to 5 L in some cases, due to the hypervascular nature of VHs [1, 51]. Acosta et al. reported an average blood loss of 2.1 L in their series of 10 aggressive VHs treated with corpectomy [51]. Conversely, laminectomy has a lower surgical burden and reduced risk of significant intraoperative blood loss [1]. Laminectomy blood loss can be further reduced by nearly 50% by performing vertebroplasty before laminectomy [8]. Preoperative embolization of VHs should also be considered to minimize intraoperative blood loss and reduce mortality [1, 22].

Goldstein et al. demonstrated that en bloc resection may not be necessary, as intralesional resection produced equivalent long-term survival and prevention of recurrence in their series of 65 patients [47]. However, there have not been any large-scale studies comparing outcomes and recurrence rates of indirect decompression versus corpectomy [1].

The treatment algorithm proposed by Teferi et al. suggests dividing symptomatic VH patients with radiculopathy or neurological deficit into cohorts of epidural spinal cord compression (ESCC) versus vertebral body compression fracture to determine appropriate surgical intervention (Fig. 6) [1]. Patients with ESCC are encouraged to undergo preoperative embolization followed by laminectomy with or without fusion depending on spinal stability, or preoperative embolization followed by corpectomy and fusion if ESCC is accompanied by extensive anterior column compromise [1]. Conversely, the recommended treatment for symptomatic VHs secondary to vertebral body compression fracture is posterior laminectomy with decompression and fusion [1].

Whether through corpectomy or laminectomy, surgical management of VHs has a low recurrence rate [1]. Piper et al. reported complete remission in 84% of VHs treated surgically in their 2020 meta-analysis [52]. They also reported a severe complication rate, including pathological fracture, significant intraoperative blood loss, wound infection, and cerebrospinal fluid leak, of 3.5% [1, 52].

### Percutaneous techniques

Percutaneous techniques include vertebroplasty, sclerotherapy, and embolization which have been rising in popularity as treatment options for VHs in isolation or in combination with surgery [1].

Vertebroplasty is a minimally invasive procedure that improves the structural integrity of a vertebra by injecting an acrylic compound, such as polymethyl methacrylate (PMMA), into a lesion [1]. It was first utilized in the treatment of VHs by Galibert et al. in 1987 [53]. PMMA causes thrombosis and irreversible sclerosis of the hemangiomatous venous pool, shrinking the lesion and consolidating trabecular microfractures [1]. It allows for rapid recovery of mobility, enhances anterior column support, and provides vertebral stabilization, but does not induce new bone formation due to poor biological activity and absorbability [54, 55]. Vertebroplasty is particularly effective in alleviating back pain in VH patients with intravertebral fractures by providing an immediate analgesic effect and has previously been recommended as stand-alone first line therapy for VHs with moderate to severe back pain without neurologic compromise [1, 54]. It can also be used in combination with surgery to reduce intraoperative blood loss when given as a preoperative adjunct therapy [8]. The most common complication of vertebroplasty is extravasation of injected compound outside the vertebral body with rates of 20–35% [55, 56]. However, some researchers suggest small amounts of extravasation should be considered a stopping point rather than a complication as the vast majority of cases are asymptomatic [55, 56]. In a series of 673 vertebroplasty cases, Layton et al. reported extravasation in 25% of patients with only 1% developing clinical symptoms of new onset radiculopathy (5 patients) or symptomatic pulmonary embolism (1 patient) [56]. Their second most common complication was rib fracture related to lying prone on the fluoroscopy table during the procedure which occurred in 1% of cases (7 patients) [56].

Alternatively, sclerotherapy involves direct intralesional injection of ethanol under percutaneous CT-guidance which causes thrombosis and destruction of endothelium, resulting in devascularization, shrinkage of the lesion, and, consequently, decompression of the neural elements [46]. It was first described as a treatment for VHs in 1994 by Heiss et al. and is less common in the treatment of VHs [57]. CT angiography is a prerequisite to target the most hypervascular subsection of the lesion and ensure patients are candidates for the procedure without leakage of contrast media, which occurred in 25% of patients in a series of 18 cases [58]. There are reports of intraoperative sclerotherapy as an adjunct to surgery, but the sample sizes are similarly limited [59, 60]. Complications of direct ethanol injection include neurologic deterioration (including Brown- Sequard syndrome), pathologic fractures, and VH recurrence [46, 61].

The last option for percutaneous intervention is trans-arterial embolization of feeding vessels using particulate agents [1]. It has been used as a preoperative adjunct therapy with surgery to reduce blood loss as well as a primary treatment for VHs alone or in conjunction with vertebroplasty [41, 62–64]. In a series of 26 patients, Premat et al. demonstrated embolization combined with vertebroplasty was safe and effective in treating pain associated with aggressive VHs but was less effective in resolving motor deficits [65]. The primary role for embolization in the treatment of compressive VHs is preoperative adjunct therapy to reduce the risk of procedural bleeding [62].

### Radiotherapy

Radiotherapy (XRT) is a noninvasive approach that can obliterate hemangiomas and relieve pain through vascular necrosis and/or anti-inflammatory effects [1]. It is a suitable option for VH patients with back pain and no neurologic deficits, or as postoperative adjunct therapy after suboptimal surgical decompression. Patients with neural element compromise often require prompt decompression to prevent irreversible injury that is more appropriately managed with surgery rather than the delayed response offered by XRT [1, 21, 66]. Neurological deficits may, in fact, be aggravated by XRT, as demonstrated in 20% of patients with aggressive VHs from a series of 29 cases by Jiang et al. [8]. Multiple studies have proclaimed a 60–80% success rate in eliminating symptoms from VHs using XRT, which increases to over 90% when including partial symptom relief [8, 67, 68]. This does include neurological deficits in some cases, but the response of these symptoms to XRT continues to vary [52]. A radiation dose of at least 34 Gy was recommended by Heyd et al. after their multicenter study identified significantly greater symptom relief and recurrence control compared to lower doses [67].

XRT is gaining popularity as a postoperative adjunct therapy intended to reduce local recurrence, especially in subtotal resections [8, 52, 67]. There is a 50% recurrence rate in partial resections without adjunct XRT [8, 11]. The extent to which XRT can reduce recurrence has not been fully elucidated and has been suggested for future study [52]. However, these potential benefits must be weighed against the known adverse effects including nausea, fatigue, anorexia, ileus, radionecrosis, and specifically in spinal XRT, radiation myelitis [1, 8, 52].

## Conclusion

VHs are often asymptomatic, incidental findings on routine spinal imaging that do not require treatment or follow-up imaging unless they become symptomatic. Most can be diagnosed with characteristic CT and MRI findings while atypical lesions may be difficult to differentiate from alternative diagnoses. Some authors suggest the utilization of emerging imaging techniques such as DWI or DCE MRI to differentiate atypical lesions from malignancies, which is a promising solution that requires further research. Other authors suggest observation with regular follow-up may be the best course of management for asymptomatic, atypical lesions while others still recommend biopsy for definitive diagnosis of atypical lesions. Regardless, there is a consensus that symptomatic lesions should be treated. Most authors recommend surgical decompression for treatment in patients with neurological deficits, but there is ongoing debate as to the optimal treatment for back pain alone. There are several treatment options which should be considered case-by-case given the properties of various lesions. Management algorithms have been suggested but additional research is required to identify the optimal treatment for the many different classifications of VHs.

## Data Availability

Data sharing is not applicable to this article as no datasets were generated or analyzed during the current study.

## Abbreviations

VH: Vertebral hemangioma
CT: Computed tomography
MRI: Magnetic resonance imaging
AV: Arteriovenous
PET-CT: Positron emission-computed tomography
^99^Tc-MDP: Technetium 99-methyl diphosphonate
DWI: Diffusion weighted imaging
ADC: Apparent diffusion coefficient
K^trans^: Volume transfer constant
DCE MRI: Dynamic contrast enhanced magnetic resonance imaging
ESCC: Epidural spinal cord compression
PMMA: Polymethyl methacrylate
XRT: Radiotherapy
